# Supplementary material for: Comparative Genomics Reveals Genetic Diversity and Variation in Metabolic Traits in Fructilactobacillus sanfranciscensis Strains
Source: Microorganisms. 2024 Apr 23;12(5):845. doi: 10.3390/microorganisms12050845 (PMC11124214; doi:10.3390/microorganisms12050845)
Supplement: Supplementary file 1 [file microorganisms-12-00845-s001.zip › microorganisms-2978193-supplementary.pdf]

**Table S1.** The carbohydrate-active enzymes genes related of exopolysaccharides (EPS) of 14 strains of *Fructilactobacillus sanfranciscensis*.

| Carbohydrate-active enzymes family | GH68 (levansucrase; EC 2.4.1.10) | GH70 (dextransucrase; EC 2.4.1.5) | GT5 (α-1,3-glucan synthase; EC 2.4.1.183) |
|---|---|---|---|
| Strains | Cleaves sucrose into fructose and glucose and use the cleavage energy to form levan out of fructose. | Transfer a D-glusosyl residue from sucrose to a glucan chain. | A glucan primer is needed to begin the reaction, which brings about elongation of the glucan chains. |
| Fs_1001 | + | - | - |
| Fs_1002 | - | - | - |
| Fs_1003 | + | + | + |
| Fs_1004 | - | + | - |
| Fs_1005 | - | + | - |
| Fs_1006 | - | - | - |
| Fs_1007 | + | - | - |
| Fs_1008 | - | + | - |
| Fs_1009 | + | - | - |
| Fs_1010 | - | + | - |
| Fs_1011 | - | - | - |
| Fs_1012 | - | - | - |
| Fs_1013 | - | - | - |
| Fs_1014 | - | - | - |

−: No carbohydrate-active enzymes genes related of EPS.

+: Containing the carbohydrate-active enzymes genes related of EPS.

**Table S2.** CRISPR-Cas system in *Fructilactobacillus sanfranciscensis*.

| Strains | DR sequence | DR length | CAS Type | CAS enzyme type |
|---|---|---|---|---|
| DSM20541/ TMW 1.53 | GTTTTAGAAGTACGTCATTCTAATGAGATTAAGAGC | 36 | IIA | cas9, cas1, cas2, csn2 |
| TMW1.54 | GTATTCCCCACGCATGTGGGGGTGATCCT | 29 | IE | cas3, cas5, cas6, cas7 |
| TMW 1.392 | GTTTTAGAAGTACGTCATTCTAATGAGATTAAGAG | 35 | IIA | cas9, cas1, cas2, csn2 |
| TMW 1.640 | GTTTTAGAAGTATGTCATTCTAATGAGATTAAGAGC | 36 | IIA | cas9, cas1, cas2, csn2 |
| | GTATTCCCCACGCATGTGGGGGTGATCC | 28 | IE | cas5, cas6, cas7 |
| TMW 1.726 | GTTTTAGAAGTACGTCATTCTAATGAGATTAAGAGC | 36 | IIA | csn2 |
| TMW 1.897 | GCTCTTAATCTCATTAGAATGACGTACTTCTAAAAC | 36 | IIA | cas2, cas1, cas9, csn2 |
| TMW 1.907 | GTTTTAGAAGTACGTCATTCTAATGAGATTAAGAGC | 36 | IIA | cas2, cas1, cas9, csn2 |
| TMW 1.936 | GCTCTTAATCTCATTAGAATGACGTACTTCTAAAAC | 36 | IIA | csn2, cas2, cas1, cas9 |
| TMW 1.1150 | GTTTTAGAAGTACGTCATTCTAATGAGATTAAGAGC | 36 | IIA | cas9, cas1, cas2, csn2 |
| TMW 1.1152 | GCTCTTAATCTCATTAGAATGACGTACTTCTAAAAC | 36 | IIA | csn2, cas2, cas1, cas9 |
| TMW 1.1154 | GCTCTTAATCTCATTAGAATGACGTACTTCTAAAAC | 36 | IIA | csn2, cas2, cas1, cas9 |
| TMW 1.1221 | GTTTTAGAAGTACGTCATTCTAATGAGATTAAGAGC | 36 | IIA | cas2, cas1, csn2 |
| TMW 1.1304 | AGGATCACCCCCACATGCGTGGGGAATAC | 29 | IE | cas5, cas6, cas7 |
| TMW 1.1470 | GTTTTAGAAGTACGTCATTCTAATGAGATTAAGAGC | 36 | IIA | cas9, cas1, cas2, csn2 |
| TMW 1.1730 | GTATTCCCCACGCATGTGGGGGTGATCCT | 29 | IE | cas3, cas5, cas6, cas7 |
| TMW 1.2137 | GCTCTTAATCTCATTAGAATGACGTACTTCTAAAAC | 36 | IIA | csn2, cas2, cas1, cas9 |
| TMW 1.2138 | GCTCTTAATCTCATTAGAATGACGTACTTCTAAAAC | 36 | IIA | cas2, cas1, cas9, csn2 |

| | | | | |
|---|---|---|---|---|
| TMW 1.2139 | GTTTTAGAAGTACGTCATTCTAATGAGATTAAGAGC | 36 | IIA | cas2, cas1, cas9, csn2 |
| TMW 1.2140 | GTTTTAGAAGTACGTCATTCTAATGAGATTAAGAGC | 36 | IIA | cas2, cas1, cas9, csn2 |
| TMW 1.2142 | GCTCTTAATCTCATTAGAATGACGTACTTCTAAAAC | 36 | IIA | csn2, cas2, cas1, cas9 |
| LS451 | GCTCTTAATCTCATTAGAATGACGTACTTCTAAAAC | 36 | IIA | csn2, cas2, cas1, cas9 |
| JCM 5668 | GCTCTTAATCTCATTAGAATGACGTACTTCTAAAAC | 36 | IIA | csn2, cas2, cas1, cas9 |
| Ls-1001 | GCTCTTAATCTCATTAGAATGACGTACTTCTAAAAC | 36 | IIA | cas2, cas1, csn2 |
| Ah4 | GCTCTTAATCTCATTAGAATGACGTACTTCTAAAAC | 36 | IIA | cas2, cas1, cas9, csn2 |
| Gs2 | GCTCTTAATCTCATTAGAATGACGTACTTCTAAAAC | 36 | IIA | csn2, cas2, cas1, cas9 |
| Gs2 | GTATTCCCCACGCATGTGGGGGTGATCC | 28 | IE | cas1, cas2, cas3, cas5, cas6, cas7 |
| Gs9 | GCTCTTAATCTCATTAGAATGACGTACTTCTAAAAC | 36 | IIA | csn2 |
| Ts9 | GTTTTAGAAGTACGTCATTCTAATGAGATTAAGAGC | 36 | IIA | cas2, cas1, csn2 |
| Sd1_3 | GTTTTAGAAGTACGTCATTCTAATGAGATTAAGAGC | 36 | IIA | cas2, cas1, cas9, csn2 |
| Fs_1001 | GTTTTAGAAGTACGTCATTCTAATGAGATTAAGAGC | 36 | IIA | cas9, cas1, cas2, csn2 |
| Fs_1001 | GTATTCCCCACGCATGTGGGGGTGATCC | 29 | IE | cas1, cas2, cas3, cas5, cas6, cas7 |
| Fs_1002 | GCTCTTAATCTCATTAGAATGACGTACTTCTAAAAC | 37 | IIA | csn2 |
| Fs_1003 | GTTTTAGAAGTACGTCATTCTAATGAGATTAAGAGC | 38 | IIA | cas2, cas1, cas9, csn2 |
| Fs_1004 | GTTTTAGAAGTACGTCATTCTAATGAGATTAAGAGC | 37 | IIA | cas2, cas1, cas9, csn2 |
| Fs_1005 | GTTTTAGAAGTACGTCATTCTAATGAGATTAAGAGC | 37 | IIA | cas9, cas1, cas2, csn2 |
| Fs_1006 | GTTTTAGAAGTACGTCATTCTAATGAGATTAAGAGC | 36 | IIA | cas2, cas1, cas9, csn2 |
| Fs_1007 | GTTTTAGAAGTACGTCATTCTAATGAGATTAAGAGC | 36 | IIA | cas9, cas1, cas2, csn2 |
| Fs_1008 | GTTTTAGAAGTACGTCATTCTAATGAGATTAAGAGC | 36 | IIA | csn2, cas3 |
| Fs_1009 | GTTTTAGAAGTACGTCATTCTAATGAGATTAAGAGC | 36 | IIA | cas9, cas1, cas2, csn2 |

| | | | | |
|---|---|---|---|---|
| Fs_1010 | GTTTTAGAAGTACGTCATTCTAATGAGATTAAGAGC | 36 | IIA | csn2, cas3 |
| Fs_1011 | GTTTTAGAAGTACGTCATTCTAATGAGATTAAGAGC | 36 | IIA | cas2, cas1, cas9, csn2 |
| Fs_1012 | GTTTTAGAAGTACGTCATTCTAATGAGATTAAGAGC | 36 | IIA | cas2, cas1, cas9, csn2 |
| Fs_1013 | GTTTTAGAAGTACGTCATTCTAATGAGATTAAGAGC | 36 | IIA | cas2, cas1, cas9, csn2 |
| Fs_1014 | GTTTTAGAAGTACGTCATTCTAATGAGATTAAGAGC | 36 | IIA | cas2, cas1, cas9, csn2 |

**Table S3.** Prediction of intact prophage regions of *Fructilactobacillus sanfranciscensis*.

| Strain | Region | Region Length | Completeness | Score | Total Proteins | Region Position | Most Common Phage | GC % |
|---|---|---|---|---|---|---|---|---|
| DSM20541 / TMW 1.53 | - | - | - | - | - | - | - | - |
| TMW1.54 | 1 | 8.5Kb | incomplete | 30 | 14 | 2220-10785 info_outline | PHAGE_Bacill_G_NC_023719 (3) | 40.01 % |
| TMW 1.392 | - | - | - | - | - | - | - | - |
| TMW 1.640 | 1 | 8.5Kb | incomplete | 10 | 9 | 134128-142642 info_outline | PHAGE_Prochl_P_SSM2_NC_006883 (4) | 38.18 % |
| | 1 | 8.3Kb | incomplete | 40 | 10 | 25184-33578 info_outline | PHAGE_Lactob_3_521_NC_048753 (1) | 35.32 % |
| TMW 1.726 | - | - | - | - | - | - | - | - |
| TMW 1.897 | - | - | - | - | - | - | - | - |
| TMW 1.907 | 1 | 43.1Kb | intact | 130 | 55 | 69802-112961 info_outline | PHAGE_Lactob_LfeSau_NC_029068 (11) | 36.64 % |
| TMW 1.936 | 1 | 8.1Kb | incomplete | 40 | 9 | 33562-41719 info_outline | PHAGE_Brevib_Davies_NC_022980 (1) | 35.85 % |
| TMW 1.1150 | - | - | - | - | - | - | - | - |
| TMW 1.1152 | 1 | 8.4Kb | incomplete | 40 | 10 | 9498-17923 info_outline | PHAGE_Brevib_Davies_NC_022980 (1) | 35.30 % |
| TMW 1.1154 | 1 | 8.4Kb | incomplete | 40 | 10 | 68950-77375 info_outline | PHAGE_Brevib_Jimmer2_NC_041976 (1) | 35.30 % |

| | | | | | | | | |
|---|---|---|---|---|---|---|---|---|
| TMW 1.1221 | 1 | 8.4Kb | incomplete | 40 | 10 | 9403-17821 info_outline | PHAGE_Entero_phiEF24C_NC_009904 (1) | 35.31% |
| TMW 1.1304 | 1 | 9Kb | incomplete | 40 | 10 | 60527-69621 info_outline | PHAGE_Lactob_3_521_NC_048753 (1) | 35.23% |
| | 1 | 6.1Kb | incomplete | 10 | 9 | 82435-88554 info_outline | PHAGE_Bacill_vB_BtS_BMBtp14_NC_048640 (2) | 31.83% |
| TMW 1.1470 | 1 | 7.7Kb | incomplete | 40 | 8 | 88429-96216 info_outline | PHAGE_Lactob_Lb338_1_NC_012530 (1) | 35.41% |
| | 1 | 32.7Kb | questionable | 70 | 21 | 45696-78484 info_outline | PHAGE_Shigel_Sf6_NC_005344 (2) | 37.40% |
| TMW 1.1730 | 1 | 9Kb | incomplete | 40 | 10 | 25818-34912 info_outline | PHAGE_Yersin_fHe_Yen9_04_NC_042116 (1) | 35.22% |
| TMW 1.2137 | - | - | - | - | - | - | - | - |
| TMW 1.2138 | - | - | - | - | - | - | - | - |
| | 1 | 8.4Kb | incomplete | 40 | 13 | 3949-12392 info_outline | PHAGE_Bacill_G_NC_023719 (3) | 43.02% |
| TMW 1.2139 | 1 | 5.7Kb | incomplete | 40 | 7 | 36-5832 info_outline | PHAGE_Brevib_Abouo_NC_029029 (1) | 34.17% |
| TMW 1.2140 | - | - | - | - | - | - | - | - |
| TMW 1.2141 | 1 | 5.7Kb | incomplete | 50 | 9 | 15541-21290 info_outline | PHAGE_Lactob_phiAT3_NC_005893 (2) | 41.39% |
| TMW 1.2142 | - | - | - | - | - | - | - | - |
| TMW | 1 | 9Kb | incompl | 40 | 10 | 60527-69621 | PHAGE_La | 35.22 |

| | | | | | | | | |
|---|---|---|---|---|---|---|---|---|
| 1.2134 | | | ete | | | info_outline | ctob_Lb338_1_NC_012530 (1) | % |
| | 1 | 8.7Kb | incomplete | 60 | 6 | 6003-14729 info_outline | PHAGE_Tenaci_PTm1_NC_049340 (2) | 30.56% |
| | 1 | 10.8Kb | incomplete | 10 | 9 | 50303-61195 info_outline | PHAGE_Bacill_G_NC_023719 (2) | 31.98% |
| TMW 1.1597 | 1 | 26.9Kb | intact | 120 | 31 | 279-27210 info_outline | PHAGE_Lactob_LF1_NC_019486 (8) | 44.01% |
| | 1 | 5.7Kb | incomplete | 20 | 13 | 105-5890 info_outline | PHAGE_Lactob_Lv_1_NC_011801 (3) | 35.00% |
| | 1 | 6.6Kb | incomplete | 50 | 11 | 180297-186935 info_outline | PHAGE_Lactob_521B_NC_048752 (2) | 35.94% |
| LS451 | 2 | 8Kb | incomplete | 40 | 8 | 242757-250847 info_outline | PHAGE_Bacill_Shanette_NC_028983 (1) | 35.83% |
| | 3 | 4.9Kb | incomplete | 40 | 8 | 1143165-1148088 info_outline | PHAGE_Lactob_Lb338_1_NC_012530 (1) | 34.26% |
| JCM 5668 | - | - | - | - | - | - | - | - |
| Ls-1001 | 1 | 8.8Kb | incomplete | 30 | 10 | 12550-21414 info_outline | PHAGE_Nodula_vB_NspS_kac65v151_NC_048756 (2) | 36.25% |
| Ah4 | 1 | 8.4Kb | incomplete | 40 | 10 | 38578-46999 info_outline | PHAGE_Burkho_BcepSauron_NC_049851 (1) | 35.31% |
| | 1 | 8.8Kb | incomplete | 40 | 11 | 34145-42963 info_outline | PHAGE_Nodula_vB_NspS_kac65v1 | 36.67% |

| | | | | | | | | |
|---|---|---|---|---|---|---|---|---|
| | | | | | | | 51_NC_048756 (2) | |
| Gs2 | 1 | 8.4Kb | incomplete | 40 | 10 | 25806-34228 info_outline | PHAGE_Achrom_Motura_NC_049849 (1) | 35.33 % |
| Gs9 | 1 | 8.8Kb | incomplete | 30 | 10 | 30074-38912 info_outline | PHAGE_Nodula_vB_NspS_kac68v161_NC_048757 (2) | 36.35 % |
| Ts9 | 1 | 8.4Kb | incomplete | 40 | 10 | 19322-27743 info_outline | PHAGE_Entero_phi92_NC_023693 (1) | 35.34 % |
| Sd1_3 | 1 | 8.4Kb | incomplete | 40 | 10 | 26439-34862 info_outline | PHAGE_Brevib_Abouo_NC_029029 (1) | 35.34 % |
| Fs_1001 | 1 | 15.7Kb | incomplete | 40 | 10 | 115688-131483 info_outline | PHAGE_Bacill_Shanette_NC_028983 (1) | 0.3509 |
| Fs_1002 | 1 | 8.4Kb | incomplete | 40 | 10 | 9432-17854 info_outline | PHAGE_Brevib_Jimmer1_NC_029104 (1) | 0.3526 |
| Fs_1003 | 1 | 8.4Kb | incomplete | 40 | 10 | 38659-47080 info_outline | PHAGE_Brevib_Jimmer2_NC_041976 (1) | 0.3538 |
| Fs_1004 | 1 | 8.4Kb | incomplete | 40 | 10 | 9933-18354 info_outline | PHAGE_Brevib_Jimmer2_NC_041976 (1) | 0.3535 |
| Fs_1005 | 1 | 15.7Kb | incomplete | 40 | 10 | 115683-131478 info_outline | PHAGE_Brevib_Osiris_NC_028969 (1) | 0.3509 |
| Fs_1006 | 1 | 8.5Kb | incomplete | 40 | 10 | 37588-46110 info_outline | PHAGE_Burkho_BcepSaruman_NC_049850 (1) | 0.3523 |

| | | | | | | | | |
|---|---|---|---|---|---|---|---|---|
| Fs_1007 | 1 | 8.3Kb | incomplete | 40 | 11 | 37360-45752 info_outline | PHAGE_Entero_EFLK1_NC_029026 (1) | 0.354 |
| Fs_1008 | 1 | 8.4Kb | incomplete | 40 | 10 | 9933-18354 info_outline | PHAGE_Entero_EFLK1_NC_029026 (1) | 0.3535 |
| Fs_1009 | 1 | 8.4Kb | incomplete | 40 | 10 | 9432-17854 info_outline | PHAGE_Entero_EfV12_phi1_NC_048087 (1) | 0.3527 |
| Fs_1010 | 1 | 8.4Kb | incomplete | 40 | 10 | 29104-37564 info_outline | PHAGE_Entero_phi92_NC_023693 (1) | 0.3537 |
| Fs_1011 | 1 | 8.4Kb | incomplete | 40 | 10 | 38659-47080 info_outline | PHAGE_Staphy_vB_SscM_1_NC_047767 (1) | 0.3538 |
| Fs_1012 | 1 | 15.7Kb | incomplete | 40 | 10 | 115683-131478 info_outline | PHAGE_Strept_BRock_NC_048650 (1) | 0.3509 |
| Fs_1013 | 1 | 8.4Kb | incomplete | 40 | 10 | 119712-128135 info_outline | PHAGE_Halovi_HGTV_1_NC_021328 (1) | 0.3534 |
| Fs_1014 | 1 | 8.4Kb | incomplete | 40 | 10 | 119716-128139 info_outline | PHAGE_Brevib_Abouo_NC_029029 (1) | 0.3534 |
